# Pentabromophenol suppresses TGF-β signaling by accelerating degradation of type II TGF-β receptors via caveolae-mediated endocytosis

**DOI:** 10.1038/srep43206

**Published:** 2017-02-23

**Authors:** Chun-Lin Chen, Pei-Hua Yang, Yu-Chen Kao, Pei-Yu Chen, Chih-Ling Chung, Shih-Wei Wang

**Affiliations:** 1 Department of Biological Sciences, National Sun Yat-sen University, Kaohsiung 804, Taiwan, ROC; 2 Doctoral Degree Program in Marine Biotechnology, National Sun Yat-sen University and Academia Sinica, Kaohsiung 804, Taiwan, ROC

## Abstract

Pentabromophenol (PBP), a brominated flame retardant (BFR), is widely used in various consumer products. BFRs exert adverse health effects such as neurotoxic and endocrine-disrupting effects. In this study, we found that PBP suppressed TGF-β response by accelerating the turnover rate of TGF-β receptors. PBP suppressed TGF-β-mediated cell migration, PAI-1 promoter-driven reporter gene activation, and Smad2/3 phosphorylation in various cell types. Furthermore, PBP abolished TGF-β-mediated repression of E-cadherin expression, in addition to the induction of vimentin expression and N-cadherin and fibronectin upregulation, thus blocking TGF-β-induced epithelial–mesenchymal transition in A549 and NMuMG cells. However, this inhibition was not observed with other congeners such as tribromophenol and triiodophenol. TGF-β superfamily members play key roles in regulating various biological processes including cell proliferation and migration as well as cancer development and progression. The results of this *in vitro* study provide a basis for studies on the detailed relationship between PBP and modulation of TGF-β signalling. Because PBP is similar to other BFRs such as polybrominated diphenyl ethers (PBDEs), additional laboratory and mechanistic studies should be performed to examine BFRs as potential risk factors for tumorigenesis and other TGF-β-related diseases.

Brominated flame retardant (BFR) phenols include pentabromophenol (PBP), 2,4,6-tribromophenol (TBP), 2,4-dibromophenol, and tetrabrominated bisphenol (TBBP). PBP, TBP, and TBBP are precursors of four nonphenolic derivatives that are also used as BFRs^1^. PBP and TBP are used for developing epoxy resins and vinyl aromatic polymers and as intermediates of polyester resins^2^. BFRs and their metabolites induce potential endocrine-disrupting effects in humans and animals^3^, in addition to being detected in human milk and blood^4^. BFRs are one of the most widely used but least understood organohalogen compounds. Molecular mechanisms underlying the toxic effects of BFRs are largely unknown. *In vitro* studies have shown that PBP and TBP and their brominated phenol congeners interact with transthyretin, a human thyroxine transport protein, competing with thyroid hormone thyroxine or with oestrogen on oestrogen receptors^5^,^6^,^7^. An *in vitro* study also revealed that TBP markedly enhanced aromatase activity, whereas 6-OH-BDE99 and 6-OH-BDE47 considerably reduced aromatase activity^8^. In the present study, we determined that PBP suppressed transforming growth factor-beta (TGF-β) signalling by accelerating TGF-β receptor degradation through caveolae-mediated endocytosis.

TGF-β superfamily proteins, including bone morphogenetic proteins, inhibins, activins, and TGF-β, regulate many physiological processes such as cell proliferation, development, and differentiation. Dysregulation of these proteins is associated with cancer development, vascular diseases, and fibrosis^9^,^10^,^11^. In a canonical pathway, binding of TGF-β to TGF-β receptors induces the assembly of type I and II TGF-β receptors (TβRI and TβRII, respectively) on the plasma membrane into heteromeric complexes for transducing signals to intracellular molecules and R-Smad proteins, including Smad2 and Smad3. Next, activated R-Smad proteins form a complex with Smad4, translocate from the cytoplasm into the nucleus, and regulate the expression of target genes. In a noncanonical pathway, TGF-β induces signal transduction through MAP kinase, phosphatidylinositol-3-kinase/AKT, and Rho-like GTPase pathways^12^,^13^,^14^. Interactions between the canonical and noncanonical pathways contribute to diverse complex cellular responses to TGF-β.

Cell surface partitioning and intracellular mechanisms underlying signal transduction by TGF-β have been extensively studied in the past few decades. Moreover, TGF-β regulation at the receptor level is being increasingly recognized. TGF-β receptor availability on the cell surface is regulated precisely and is a vital determinant of cellular response to TGF-β^15^. Our and other previous studies have demonstrated that cellular responses to TGF-β are determined by TGF-β partitioning between clathrin- and caveolae-mediated endocytic pathways^16^,^17^,^18^,^19^,^20^,^21^,^22^. Clathrin-mediated endocytosis promotes signalling and cellular responses, whereas caveolae-mediated endocytosis engenders the rapid degradation of TGF-β-bound TGF-β receptors and attenuation of TGF-β response^16^,^17^,^18^,^19^,^20^,^21^,^22^. Caveolae-/lipid raft-mediated endocytosis is a common mechanism for receiving signals from an extracellular environment. Lipid rafts are membrane microdomains enriched with sphingolipids and cholesterol. Recently, lipid rafts have been extensively studied in the endocytosis of several receptors including epidermal growth factor receptor^23^, vascular endothelial growth factor receptor^24^, and G-protein coupled receptors^25^. In addition, caveolae-mediated endocytosis plays a major regulatory role in TGF-β signalling, especially in TGF-β receptor ubiquitination and degradation^26^. Clathrin-mediated endocytosis is involved in TGF-β signalling at the coated-pit stage of endocytosis^27^.

In the present study, we determined that PBP suppressed TGF-β signalling in mink lung epithelial cells (Mv1Lu cells), mouse mammary gland epithelial cells (NMuMG cells), and human lung adenocarcinoma cells (A549 cells). The *in vitro* experiments demonstrated that PBP suppresses TGF-β-induced PAI-1 promoter activation through Smad2 and Smad3 (Smad2/3) phosphorylation. In A549 and NMuMG cells, PBP significantly attenuated TGF-β-induced epithelial–mesenchymal transition (EMT), including reduction of cell migration, as well as decreased expression of EMT-related gene, such as N-cadherin, vimentin, and fibronectin. On the basis of the results of studies that have implicated the role of PBP in TGF-β receptor endocytosis and rapid degradation, we hypothesized that PBP displaces TGF-β receptors on the cell surface and facilitates their accumulation in lipid-raft membrane domains and caveolin-positive vesicles, leading to proteasome-mediated degradation and subsequent reduced TGF-β signalling. To the best of our knowledge, ours is the first study to demonstrate that pentabromophenol inhibits TGF-β responsiveness. Therefore, the present study emphasizes the potential ecotoxic and endocrine-disrupting effects of PBP in TGF-β-related diseases.

## Materials and Methods

### Materials

Fetal calf serum (FCS), 4,6-diamidino-2-phenylindole (DAPI), Alexa Fluor^®^ 488 and Alexa Fluor^®^ 594 conjugated secondary antibodies were purchased from ThermoFisher (Waltham, MA). TRIzol reagent was purchased from Invitrogen, (Carlsbad, CA). M-MLV Reverse Transcriptase was obtained from Promega (Madison, WI). Pentabromophenol (PBP), triiodophenol (TIP), bovine serum albumin (BSA), Dulbecco’s modified Eagle’s medium (DMEM), peroxidase-conjugated anti-rabbit IgG, MG132, phenylmethanesulfonyl fluoride (PMSF), ammonium chloride (NH_4_Cl), trifluoperazine (TFP), and methyl-β-cyclodextrin (MβCD) were purchased from Sigma-Aldrich (St. Louis, MO). The pre-stained protein ladder (125,93,72,57,42,31,24, and 15 kDa) was obtained from GeneDireX (Carlsbad, CA). TGF-β was obtained from PeproTech (Rocky Hill, NJ). The anti-early endosome antigen 1 (EEA1), anti-Smad2/3, anti-HA-probe, anti-caveolin-1, anti-flotilin-2, anti-TβR-I, and anti-TβR-II polyclonal antibodies were obtained from Santa Cruz (Dallas, TX). The rabbit polyclonal antibody to phospho-Smad2 was purchased from Cell Signaling (Boston, MA). A mink lung epithelial cell line (Mv1Lu cells, CCL-64) was a gift from Dr. Jung San Huang from Saint Louis University. A549 cells (human lung adenocarcinoma cell, CCL-185) were purchased from ATCC (Manassas, VA). All cell lines in this study were maintained in DMEM containing 50 μg/ml streptomycin and 5% FCS. NMuMG cells (mouse mammary gland epithelial cell, CRL-1636, ATCC) were cultured in DMEM containing 50 μg/ml, streptomycin 5% FBS and supplied with 10 μg/ml insulin. PAI-1 promotor stable clone of Mv1Lu cells (MLECs-Clone 32) was a gift from Dr. Jung San Huang in Saint Louis University. The *COL1A2-luc* plasmid was constructed as described by Poncelet *et al*.^28^. The *Fibro-luc* plasmid was constructed as described by Cobbs and Widom^29^,^30^. The stock solution of PBP (20 mM) was prepared in EtOH. The final concentrations of EtOH in all experiments were lower than 0.1% which has no effect in TGF-β signaling^31^.

### Cell surface TGF-β receptor biotinylation and endocytosis assays

Surface biotinylation was performed at 0 °C using 0.2 mM Sulfo-NHS-SS-biotin (ThermoFisher) according published procedures^32^. Biotinylated cell lysates were analyzed by 10% SDS-PAGE followed by immunoblotting analysis and quantification using ImageQuant. Mv1Lu cells grown to 90% confluence on 6-well cluster plates were treated with PBP for different time periods at 37 °C. After treatment, cells were washed with cold PBS and incubated with 0.2 mM Sulfo-NHS-SS-biotin for 30 min. Biotinylated cells were washed with TBS and the cells then were lysed in lysis buffer and incubated with streptavidin beads for 1 h at 4 °C. Strptavidin-precipitated TβRII protein was detected using immunoblotting. The biotinylated TβRII remaining on the cell surface should be compared to the total TβRII level before biotinylation.

### Analysis of lipid raft/caveolae and non-lipid raft microdomains

To separate and analyze the membrane microdomains, we performed sucrose density gradient ultracentrifugation according published procedures^33^ without any modification. Mv1Lu were grown on 100 mm dishes (5 × 10^6^ cells per dish). Cells were then incubated with or without 5 μM PBP in low serum (0.1% FBS) DMEM at 37 °C for the time indicated^18^. After two washes with ice cold phosphate-buffered saline, cells were scraped into 0.85 ml of 500 mM sodium carbonate, pH 11.0. Homogenization was carried out by three 15-second bursts of an ultrasonic disintegrator (Qsonica, Newtown, CT, USA) to disrupt cell membranes, as described previously^18^. The homogenates were adjusted to 45% sucrose by addition of 0.85 ml of 90% sucrose in 25 mM 2-(N-morpholino) ethanesulfonic acid, pH 6.5, 0.15 M NaCl (MBS), and placed at the bottom of an ultracentrifuge tube. A discontinuous sucrose gradient was generated by overlaying 1.7 ml of 35% sucrose and 1.7 ml of 5% sucrose in MBS on the top of the 45% sucrose solution, and it was then centrifuged at 40,000 rpm for 16–20 h in an SW55 TI rotor. Ten 0.5-ml fractions were collected from the top of the tube, and a portion of each fraction was analyzed by immunoblotting using antibodies against TβRII. The relative amounts of TβRII on the blot were quantified by densitometry. Fractions 4–5, and fractions 7 to 10 contained flottlin-2 and EEA-1, respectively^18^,^33^.

### Immunoblotting analysis analysis

Cell lysates (~50 μg protein) were subjected to 7.0%, 10%, or 12.5% SDS-PAGE under reducing conditions and then electrotransferred to PVDF membranes. After being incubated with 5% nonfat milk in Tris-buffered saline plus Tween 20 (TBST) (50 mM Tris-HCl, pH 8.0, 150 mM NaCl, 0.05% Tween 20) for 1 h at room temperature, the membranes were further incubated with specific polyclonal antibodies to TβR-I and TβR-II in TBST/non-fat milk at 4 °C for 20 h and washed three times with TBST for 10 min each. Bound antibodies were detected using peroxidase-conjugated anti-rabbit or anti-mouse IgG and visualized using the ECL system.

### Immunofluorescent staining

To determine the effect of PBP in TGF-β-induced EMT, cells on 24 mm round coverslips (Paul Marienfeld, Germany) were pretreated with or without 2 μM PBP for 2 h in low serum DMEM (0.1% FBS), cells were then continuingly stimulated with TGF-β (100 pM) for 48 h. Treated cells were washed with phosphate buffered saline (PBS) and fixed in cold methanol for 10 min. After washings with PBS, cells were blocked with 5% goat serum (Dako) in 1% BSA/PBS. After incubation with rabbit anti-E-cadherin, anti-vimentin, anti-N-cadherin, and anti-fibronectin antibodies (1:200) in 1% BSA/PBS for 18 h at 4 °C, cells were incubated with donkey anti-rabbit-Alexa Fluor^®^ 488 at RT for 1 h. Coverslips were mounted with mounting medium containing DAPI (ThermoFisher). Photomicrographs were taken with a Zeiss Axio Observer Z1 microscope equipped with a Photometrics HQ2 camera.

To determine the effect of PBP in subcellular localization of TβRII, Mv1Lu cells grown on 24 mm round coverslip were transiently co-transfected with TβRII-HA and caveolin-1-GFP plasmids using Lipofectamin 2000 (ThermoFisher) according to the manufacturer’s protocol. Twenty-four hours after transfection, cells were changed to low serum medium (0.1% FBS) and treated with PBP 5 μM for the time indicated. After treatment, cells were fixed in 4% paraformaldehyde solution containing 0.1% Triton-X100 for 30 minutes, washed with PBS and then blocked by 0.2% gelatin in PBS for 1 h. Cells were incubated overnight at 4 °C in a humidified chamber with a goat anti-HA-probe (F-7; Santa Cruz Biotechnology) at 1:100 dilutions. After extensive washing, cells were incubated with Alexa Fluor^®^ 594-conjugated donkey anti-goat antibody at a 1:50 dilution for 1 h. Images were acquired using a Nikon TCS SP confocal microscope (Nikon Ltd., Tokyo, Japan). The measurements of co-localization rate were analyzed using a Nikon Application Suite.

### Transcriptional response assay

The procedurals for transcriptional assay were performed in Mv1Lu or MLE cells according to our recent report^21^,^34^ and are described concisely as follow. Mv1Lu cells were transiently transfected with CMV-βgal, and *Fibro-luc*^35^ or *COL1A2-luc*^35^ reporter plasmids using electroporation. In a similar experiment, MLE cells (Mv1Lu cells stably expression 3TP-luc promoter plasmid) were also used. Cells grown in low serum medium were incubated with several concentrations of PBP for 1 h follow by TGF-β treatment for 4 h. Fifty micro liter cell lysates (approximately 20 μg of protein) were then used to measure both luciferase and β-gal activities. The luciferase activity was normalised and the increment of luciferase activity was calculated against the experimental controls^21^.

### Scratch wound assay

The procedural for cell migration assay was descripted in our previous work^34^. Briefly, A549 cells grown in 4-chambered 35-mm dish (95% confluency) were serum-starved in DMEM containing 0.1% FBS for 2 h prior to wounding to ensure that no proliferation occurred during the experiments. A scratch wound was created by using a 200 μl pipette tip on cells monolayers. The wounded cells were immediately treated with TGF-β (100 pM) in the presence or absence of 5 μM PBP for 15 h. Digital images of the cells that had migrated into the wound area were taken by an Axio Observer Z1 inverted microscope fitted with a K heating stage and incubator (Carl Zeiss Inc., Oberkochen, Germany).

### Statistical Analysis

All experiments were conducted in triplicate. All data were shown as the mean ± standard deviation (S.D.). We used Student’s t test for the comparison between two groups, and used One-way ANOVA when we compared more than two groups. The means were considered significant if *P* < 0.05 (*) or *P* < 0.01 (**).

## Results

To rule out the cytotoxicity effects mediated by PBP in this study, we performed the toxicity assays and cell viability assays by testing plasma membrane integrity and mitochondria functions (i.e., MTT assay). Acute toxicity of PBP was determined by measuring G6PDH leakage (data not shown), and the IC_50_ of PBP on NMuMG cells and A549 cells are more than 30 μM (Figure S1). Therefore, the doses of PBP used were between 1 μM and 5 μM in subsequent experiments.

### The TGF-β-induced Smad phosphorylation and promoter activation were suppressed by PBP in cells

**Figure 1:**
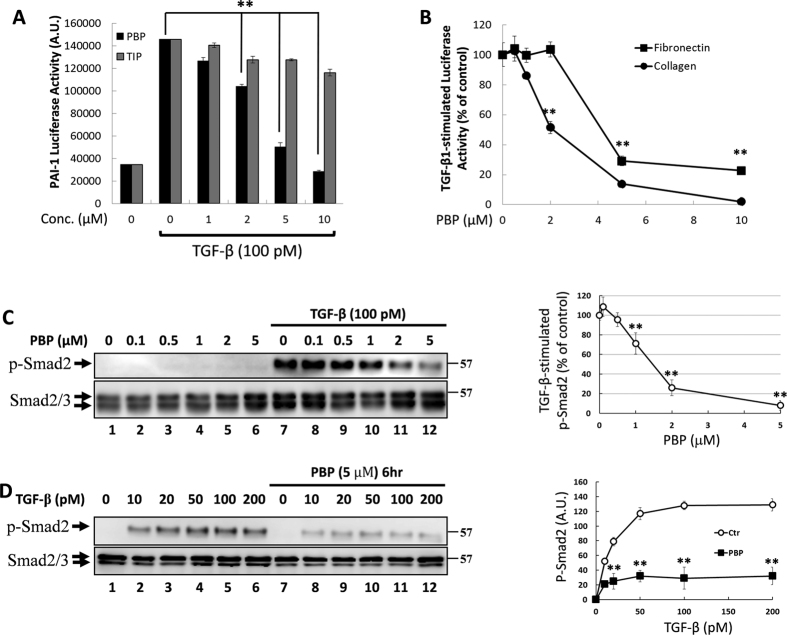
PBP blocked TGF-β signalling *in vitro*. (**A**) PBP abolished TGF-β-stimulated transcriptional activity in a dose-dependent manner. Mv1Lu cells stably expressing the luciferase reporter gene under the control of the PAI-1 promoter (MLE cells clone 32) were grown in 24-well cluster plates, incubated with TGF-β in the presence or absence of PBP or 2,4,6-triiodophenol (TIP), and then analysed by performing a luciferase assay. (**B**) Mv1Lu cells were transiently transfected with fibronectin (*Fibro-luc*) and collagen (*COL1A2-luc*) luciferase promoter plasmids and then analysed by performing the luciferase assay. (**C** and **D**, right graph for quantification) Mv1Lu cells were treated with PBP for 6 h, and cell lysates were resolved by performing immunoblotting analysis to assess Smad2 phosphorylation. Smad2/3 served as an internal control. All experiments were repeated three times, and data are expressed as mean ± SD. Dual asterisks indicate significant differences (*P* < 0.01), as determined using one-way analysis of variance with SPSS statistical software.

TβRI and TβRII are expressed in all normal cells, but they are not expressed in some cancer cells^9^,^10^,^11^. In the presence of TGF-β, TβRI and TβRII form a hetero-oligomeric complex that activates canonical (Smad-dependent) and noncanonical (Smad-independent) TGF-β signalling and is crucial for many cellular processes including cell growth, apoptosis, differentiation, extracellular matrix production, and EMT^9^,^11^,^13^. Mv1Lu cells have been widely used as a model for studying TGF-β signalling and relative cell responses^16^,^17^,^18^,^19^. Mv1Lu cells expressing 3TP-Lux luciferase promotor (termed as MLE cells) were used to evaluate the inhibitory effects of PBP in TGF-induced cellular responses. 3TP-Lux contains three consecutive TPA response elements (TREs) and a portion of the PAI-1 promoter region. In Fig. 1A, TGF-β stimulation resulted in a fivefold increase of luciferase activity in MLE cells harbouring the 3TP-Lux luciferase promoter. PBP attenuated TGF-β-induced luciferase activity in a dose-dependent manner, with the IC_50_ value of PBP being approximately 3 μM and the maximum inhibition being achieved using 10 μM PBP (Fig. 1A, black columns). However, a structurally related congener of PBP, such as 2,4,6-triiodophenol (TIP), did not considerably affect TGF-β-stimulated PAI-1 promoter activity in MLE cells (Fig. 1A, grey columns). In addition to PAI-1 gene, collagen type I, α2 and fibronectin genes are also important targets for canonical TGF-β signalling^28^,^29^,^30^. Mv1Lu transiently expressing *COL1A2-luc* or *Fibro-luc* were used to determine the effects of PBP on TGF-β signalling and β-galactosidase expression serving as an internal control. Figure 1B shows that TGF-β-induced transcription of collagen and fibronectin were inhibited by PBP in a dose-dependent manner. To further determine the specific target of PBP on canonical (Smad-dependent) TGF-β signalling, we performed immunoblotting to observe the levels of phosphorylated Smad2 in Mv1Lu cells treated with PBP in the presence of TGF-β. Smad2/3 proteins are the major signal transducers of TGF-β signalling. TGF-β stimulation activates Smad2/3 by phosphorylation at their C-terminal serine residues through TβRI–TβRII receptor complexes. Next, phosphorylated Smad2/3 complexes with Smad4 migrate into the nucleus and activate various target genes. In this study, Mv1Lu cells were pretreated with increasing concentrations (0 to 5 μM) (Fig. 1C) or single concentration (5 μM) (Fig. 1D) of PBP for 6 h, followed by TGF-β stimulation for 30 min. TGF-β treatment strongly stimulated Smad2 phosphorylation in Mv1Lu cells; however, PBP pretreatment inhibited TGF-β-induced Smad2 phosphorylation in a dose-dependent manner in Mv1Lu cells (Fig. 1C), with the IC_50_ value of PBP being approximately 1.5 μM and the maximum inhibition being achieved using 5 μM PBP (Fig. 1C, lanes 7–12, right graph for quantification). The level of phosphorylated Smad2 increased in a dose-dependent manner in the Mv1Lu cells treated with increasing concentrations of TGF-β (Fig. 1D, lanes 1–6); nevertheless, PBP treatment inhibited the Smad2 phosphorylation induced by all concentrations of TGF-β used (Fig. 1D, lanes 8–12, right graph for quantification). This finding was also validated in NMuMG cells, suggesting that PBP inhibits TGF-β-induced Smad phosphorylation, regardless of the cell type (Supplemental data, Figure S2).

### PBP attenuates TGF-β-induced EMT

**Figure 2:**
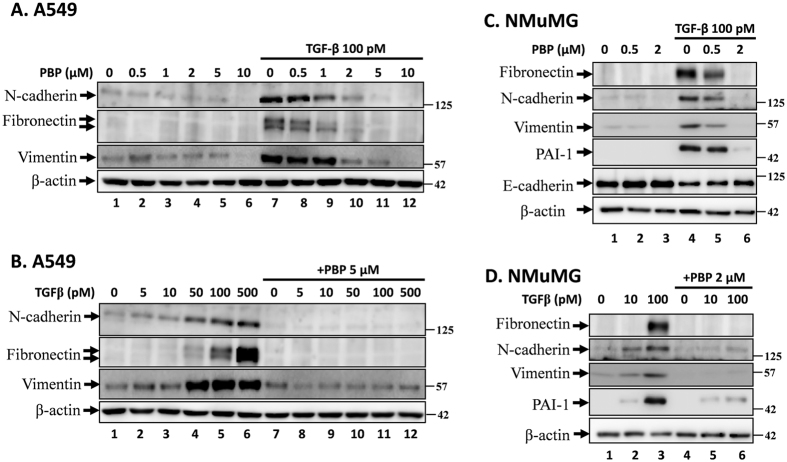
PBP attenuated TGF-β-induced EMT and fibronectin and PAI-1 expression in A549 and NMuMG cells. A549 (**A**) and NMuMG (**C**) cells were treated with 100 pM TGF-β in the presence of increasing concentrations of PBP for 48 h. Cell lysates were resolved by performing immunoblotting with specific antibodies against fibronectin, N-cadherin, vimentin, E-cadherin, PAI-1, and β-actin. A549 (**B**) and NMuMG (**D**) cells were treated with increasing concentrations of TGF-β or PBP for 48 h.

**Figure 3:**
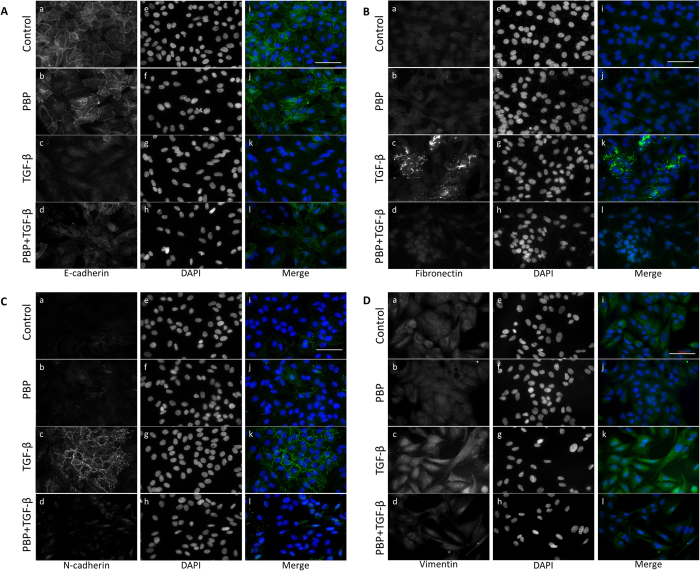
PBP attenuated TGF-β-induced EMT in A549 cells. EMT was determined by immunostaining for epithelial marker E-cadherin (**A**), ECM protein fibronectin (**B**), and mesenchymal markers N-cadherin and vimentin (**C** and **D**, respectively). A549 cells cultured on a cover glass were treated with TGF-β (100 pM) in 0.1% FCS in the presence or absence of PBP (5 μM) for 48 h. Cells were fixed with 4% paraformaldehyde and then incubated with primary antibodies against E-cadherin, fibronectin, N-cadherin, and vimentin. Fluorescence signals were visualised using Alexa Fluor 488-conjugated secondary antibodies. Nuclei of the cells were stained with DAPI. Scale bar = 200 μm.

TGF-β–Smad signalling strongly induces EMT^36^. NMuMG and A549 cells have been extensively used as *in vitro* models for studying EMT, and these cells undergo EMT discernible at 40 h after TGF-β stimulation^37^,^38^. To understand whether PBP could suppress TGF-β-induced EMT, expression of EMT markers including fibronectin, vimentin, N-cadherin, and E-cadherin in A549 and NMuMG cells were evaluated by immunoblotting analysis and immunofluorescence staining. EMT is characterised by E-cadherin disruption from cell junctions and by increased fibronectin, N-cadherin, and vimentin expression^39^. In immunoblotting analysis, the A549 and NMuMG cells were pre-treated with increasing concentrations (Fig. 2A and C) or a single concentration (5 μM and 2 μM PBP for the A549 cells and the NMuMG cells, respectively) of PBP for 2 h, followed by TGF-β stimulation for 48 h. TGF-β treatment increased the expression levels of N-cadherin, fibronectin, and vimentin in the A549 (Fig. 2A, lane 1 versus lane 7, and 2B, lane 1 to lane 6) and NMuMG cells (Fig. 2C, lane 1 versus lane 4 and 2D, lane 1 to lane 3) and reduced the expression of E-cadherin in the NMuMG cells (Fig. 2C, lane 1 versus lane 4). By contrast, PBP inhibited TGF-β-stimulated fibronectin, N-cadherin, vimentin, and PAI-1 protein expression in a dose-dependent manner (Fig. 2A, lanes 7 to 12); 5 μM PBP completely eliminated the expression of N-cadherin, fibronectin, and vimentin stimulated by increasing concentration of TGF-β in A549 cells (Fig. 2B, lanes 7–12). In NMuMG cells, PBP inhibited TGF-β-stimulated fibronectin, N-cadherin, vimentin, and PAI-1 protein expression in a dose-dependent manner (Fig. 2C, lanes 4–6); conversely, PBP treatment slightly reversed the TGF-β-induced E-cadherin disruption by 18% (Fig. 2C, lane 4 versus lane 6). In addition, 2 μM PBP inhibited TGF-β-induced fibronectin, N-cadherin, vimentin, and PAI-1 protein by more than 90% (Fig. 2D, lanes 4 to 6). Consistent with the immunoblotting results, the immunofluorescence staining results revealed that PBP reversed TGF-β-induced suppression of E-cadherin expression (Fig. 3Al versus 3Ak) and reduced TGF-β-stimulated induction of fibronectin, N-cadherin, and vimentin expression (Fig. 3Bl versus 3Bk, 3Cl versus 3Ck, and 3Dl versus 3Dk). Taken together with prior results in Fig. 1, PBP could suppress TGF-β-induced Smad phosphorylation, and causing the inhibition of EMT.

### PBP inhibits TGF-β-induced cell migration

**Figure 4:**
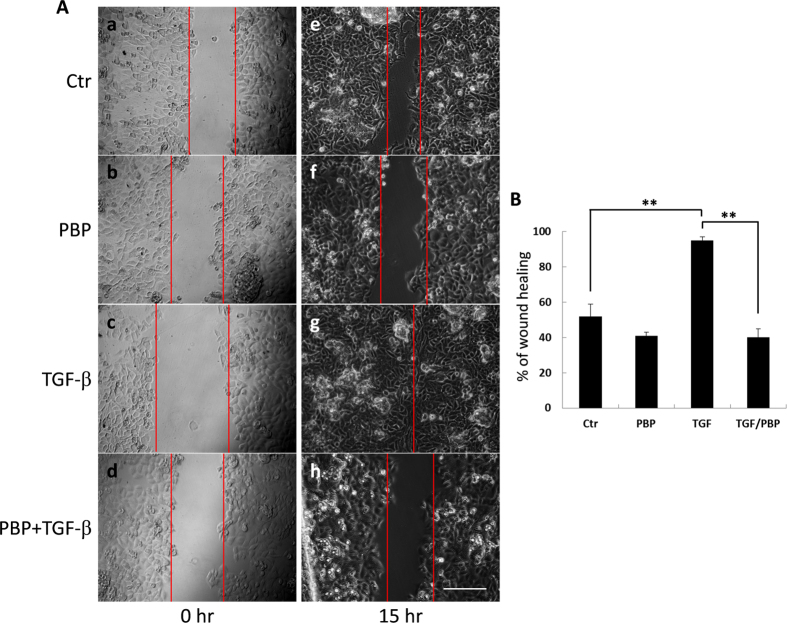
PBP inhibits TGF-β-induced motility of A549 cells. (**A**) A549 cells grown on a 4-chambered 35-mm dish were starved in DMEM containing 0.1% FCS for 12 h before wounding. Wounded cells were treated with TGF-β (100 pM) ± PBP (5 μM) for the indicated time. Cell motility was measured using a phase-contrast microscope at 100 × magnification. Cell migration was observed by performing time-lapse microscopy and imaging at 0 and 15 h after wounding. (**B**) Percentage of wound closure was calculated from the mean ± SD (error bars) of eight wound widths per condition measured at 15 h. One representative experiment out of three independent experiments is shown (**P < 0.01); scale bar = 200 μm.

In addition to inducing EMT in epithelial cells, TGF-β plays a crucial role in promoting cancer cell migration and invasion via a Smad-dependent pathway^39^,^40^. Inhibition of TβR-I with SB431542 has been shown to inhibit the function of TGF-β in cell migration^41^. To test if PBP inhibited TGF-β-stimulated cell migration, we determined the effect of PBP on TGF-β-induced increases in cell motility by performing a wound healing assay, as described previously by Lamouille *et al*.^39^,^42^. We observed that TGF-β-stimulated migration of A549 cells by inducing exhibited >95% wound closure (Fig. 4Ag) after 15 h treatment. In contrast, 5 μM PBP strongly inhibited TGF-β-induced migration of A549 cells from 95% to 42% (Fig. 4Ag versus 4Ah, and 4B). In the experiment with PBP alone, PBP reduced the percentage of wound closure from 55% to 42% (Fig. 4Ae versus 4Af, and 4B). This result indicates that PBP suppresses TGF-β-induced cell migration. It is worth noting that A549 cell is responsive to TGF-β in both cell growth and wound healing. Our [^3^H]-Thymidine incorporation assays and cell counting results (data not shown) show that A549 cells are growth-inhibited by approximately 50% and 35%, respectively. These suggest that proliferation is not involved in the migration of A549 cells induced by TGF-β. Furthermore, the results of MTT assay (Figure S1C) show that 10 μM PBP enhances cell viability by 40%, this suggest that the migratory inhibition of PBP is not due to cytotoxicity.

### PBP accelerates the internalisation of TβRII and results in its rapid degradation

**Figure 5:**
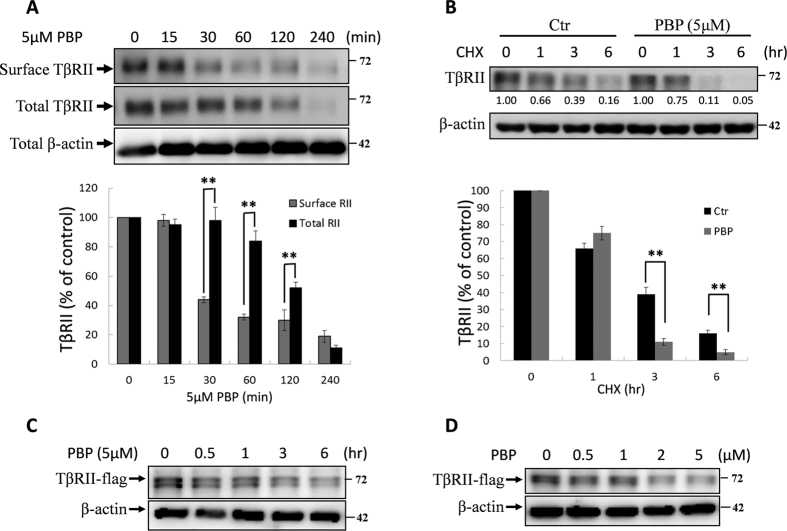
PBP-induced TβRII degradation. (**A**) PBP reduced the cell surface expression and induced rapid degradation of TβRII, as determined by conducting cell surface biotinylation and Western blotting, respectively, on Mv1Lu cells. Cells were treated with 5 μM PBP at 37 °C for 0, 0.5, 1, 3, and 6 h. Cell surface TβRII was biotinylated and pulled down using streptavidin-Sepharose beads, and it was subsequently assessed through immunoblotting analysis. Total TβRII expression was also determined. One representative experiment out of four independent experiments is shown. Relative TβRII level in cells not treated with PBP was set as 100% (graph in the lower panel). At 0.5 h, PBP reduced the cell surface and total expression of TβRII by 80% and 40%, respectively. At 6 h, PBP reduced the cell surface and total expression of TβRII by >90%. (**B**) Mv1Lu cells were treated with cycloheximide (CHX) for the indicated time in the presence or absence of PBP. Band intensity was quantified, and statistical analyses of four independent experiments are provided. (**C** and **D**) Mv1Lu cells expressing TβRII-flag were treated with PBP in the indicated time ant concentration. The cell lysates were then analyzed by immunoblotting analysis using anti-flag, β-actin antibodies.

In the preceding sections, PBP attenuated TGF-β-stimulated cellular response including reporter gene activation, Smad2 phosphorylation, and EMT. These findings prompted us to investigate the detailed mechanism underlying the inhibitory effect of PBP on TGF-β. We conjectured that PBP may reduce TGF-β activity by increasing the endocytosis and degradation of TGF-β receptors. To test this conjecture, we examined the effect of PBP on the expression of TGF-β receptors on the surface of Mv1Lu cells by performing cell surface biotinylation. The Mv1Lu cells were pretreated with 5 μM PBP for 0–2 h. At the indicated time, the cells were cooled rapidly, and proteins expressed on the surface of these cells were biotinylated. Biotinylated TβRII was pulled down by using streptavidin-Sepharose beads and was examined through immunoblotting. To determine whether PBP altered TGF-β receptor stability, we performed a parallel experiment by measuring the total receptor protein levels in the lysates of the cells treated with PBP. As expected, PBP treatment reduced TβRII protein levels both on the cell surface and in the cell lysates in a time-dependent manner (Fig. 5A). The reduction of TβRII in cell surface was started at 15–30 min (Fig. 5A, lanes 2 and 3) and started from 60–120 min for total lysates (Fig. 5A, lanes 4 and 5). Since the PBP-induced disappearance of TβRII in cell surface was faster than in whole cell lysates, which suggest that PBP-induced TβRII internalisation is prior to its degradation. However, PBP treatment did not alter the mRNA levels of TβRII (Supplemental Data Figure S3). These results signify that PBP may reduce TβRII stability. To assess the effect of PBP on TβRII stability, we monitored TβRII turnover after the impeding of protein synthesis by cycloheximide and found that PBP reduced the half-life of TβRII in the Mv1Lu cells (Fig. 5B). To further confirm that PBP accelerates TβRII turnover, Mv1Lu cells expressing TβR-II-flag were treated with 5 μM PBP for increasing time period or with increasing concentration of PBP for 4 h and were further detected by immunoblotting with the anti-flag antibody. As shown in Fig. 5C and D, PBP treatment enhanced TβR-II-flag degradation in both time- and dose-dependent manners.

**Figure 6:**
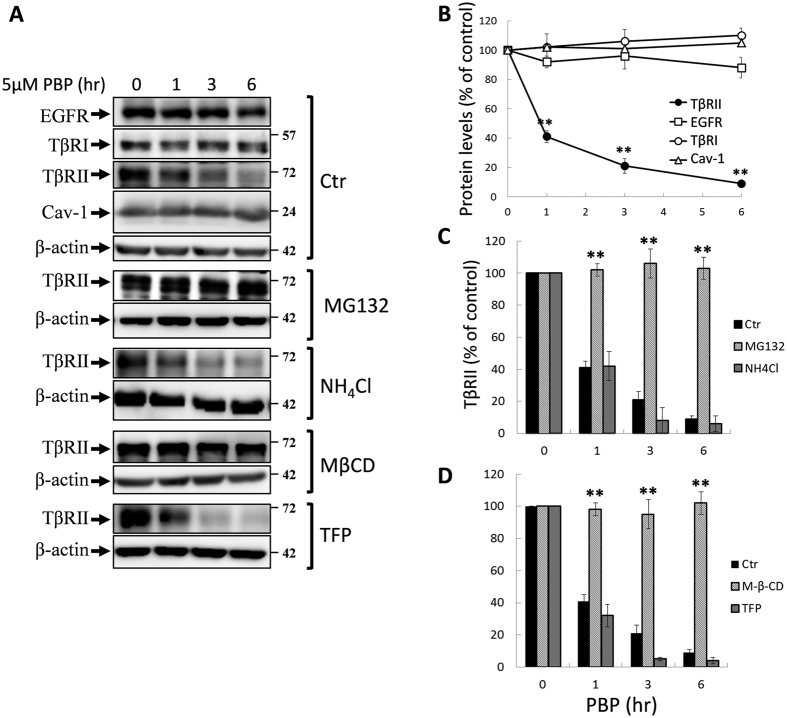
MG132 and MβCD reversed PBP-induced TβRII degradation. Mv1Lu cells treated with PBP were coincubated with MG132 (10 μM), NH_4_Cl (20 mM), MβCD (2.5 mg/ml), and TFP (20 μM) at 37 °C for the indicated time. Next, the cells were harvested and their normalised protein samples assessed through SDS-PAGE and immunoblotting with antibodies against TβRI, TβRII, EGFR, and caveolin-1 (Cav-1) (**A**). Graphs represent mean ± SD densitometry data from three independent experiments. (**B**) PBP induced the rapid degradation of TβRII but did not exert any effect on TβRI, EGFR, and caveolin-1. Dual asterisks indicate significant differences (*P* < 0.01) in comparisons between TβRII and TβRI and EGFR. (**C**) MG132, a proteasome inhibitor (but not NH_4_Cl, a lysosome inhibitor), abolished PBP-induced TβRII degradation. (**D**) MβCD, an inhibitor of caveolae-mediated endocytosis (but not TFP, an inhibitor of clathrin-mediated endocytosis), abolished PBP-induced TβRII degradation.

Because PBP enhances TβRII turnover, and it has been recognized that TβRII turnover is dynamically regulated by clathrin vesicle-mediated ligand-triggered trafficking, recycling, and lysosome degradation, as well as caveolae vesicle-mediated proteasomal degradation^19^. We used lysosomal inhibitor NH_4_Cl and proteasome inhibitor MG132 to determine the pathways involved in TβRII degradation. Our results showed that MG132 (but not NH_4_Cl) reversed PBP-induced TβRII degradation (Fig. 6A and C for quantification), signifying that proteasome-dependent degradation was primarily involved in PBP-induced TβRII degradation. Notably, PBP induced TβRII degradation without altering the EGFR, TβRI, and Cav-1 levels (Fig. 6A and B for quantification). Because TβRII was targeted to the proteasome, we examined ubiquitination of TβRII but found no evidence of mono- or polyubiquitination (Figure S5). Taken together, the PBP class of molecules comprises selective TGF-β inhibitors that function by diverting TβRII to the proteasome through an ubiquitin-independent mechanism.

### Lipid rafts/Caveolae are essential for PBP-induced TβRII degradation

**Figure 7:**
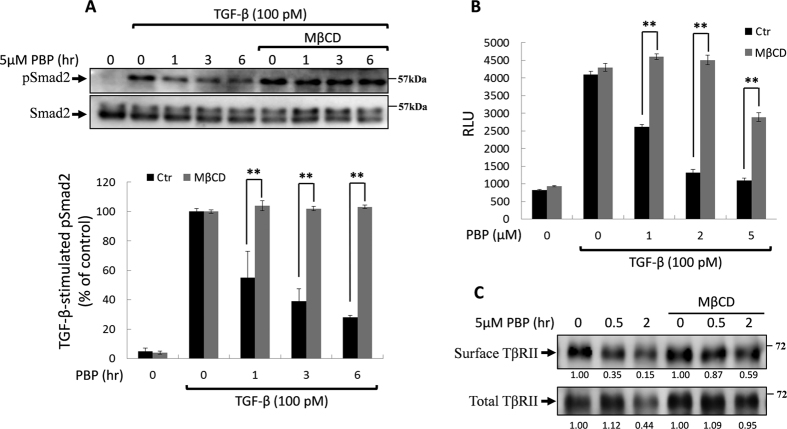
MβCD reversed PBP-induced inhibition of TGF-β signalling. Mv1Lu cells were treated with 5 μM PBP for various times in the presence or absence of MβCD, followed by stimulation with TGF-β (30 min and 4 h for Smad2 phosphorylation and PAI-1 promoter activation, respectively). (**A**) PBP inhibited TGF-β-induced Smad2 phosphorylation, which was reversed after MβCD treatment (black bars versus grey bars in the lower graph). (**B**) PBP inhibited TGF-β-induced PAI-1 promoter activation, which was reversed after MβCD treatment. Graphs represent mean ± SD densitometry data from three independent experiments. Dual asterisks indicate significant differences (P < 0.01) in comparisons between control and MβCD treatment groups.

TβRII is internalised through both caveolae- and clathrin-mediated endocytosis^32^,^43^, and caveolae-mediated endocytosis attenuates TGF-β signalling by promoting TβRII degradation. These two endocytic pathways are maintained in a dynamic balance and the inhibition of one these pathways leads to the promotion of the other pathway^19^,^32^. Methyl-β-cyclodextrin (MβCD) and trifluoperazine (TFP) were used to inhibit lipid raft/caveolae- and clathrin-mediated endocytosis, respectively^27^. We observed that PBP induced TβRII internalisation and degradation mainly through lipid raft-/caveolae-mediated endocytosis and that PBP-induced TβRII internalisation and degradation was inhibited by MβCD, rather than TFP (Fig. 6A and D for quantification). Consistent with this finding, treatment with MβCD, a cholesterol chelator and lipid raft disruptor, reversed PBP-inhibited TGF-β signalling including Smad2/3 phosphorylation (Fig. 7A, lower graph for quantification) and PAI-1 promoter activation (Fig. 7B). By contrast, treatment with TFP, the inhibitor of clathrin-mediated endocytosis, did not reverse the PBP-inhibited TGF-β signalling (data not shown). To define the chronologic sequence of TβRII internalisation and degradation after PBP treatment, we treated cells with PBP and/or MβCD follow by cell surface biotinylation. If PBP-induced TβRII degradation is secondary to its internalisation, the inhibitors of caveolae- mediated endocytosis will alleviate PBP-induced TβRII internalisation and degradation. Figure 7C indicates that MβCD, a caveolae disruptor, not only retain TβRII in the cell surface but also inhibits TβRII degradation. Echoing with prior result in Fig. 5A, this result also suggests that the proceeding of TβRII internalisation is prior to degradation.

### PBP enhance TβRII internalisation and degradation via caveolae-mediated endocytosis

**Figure 8:**
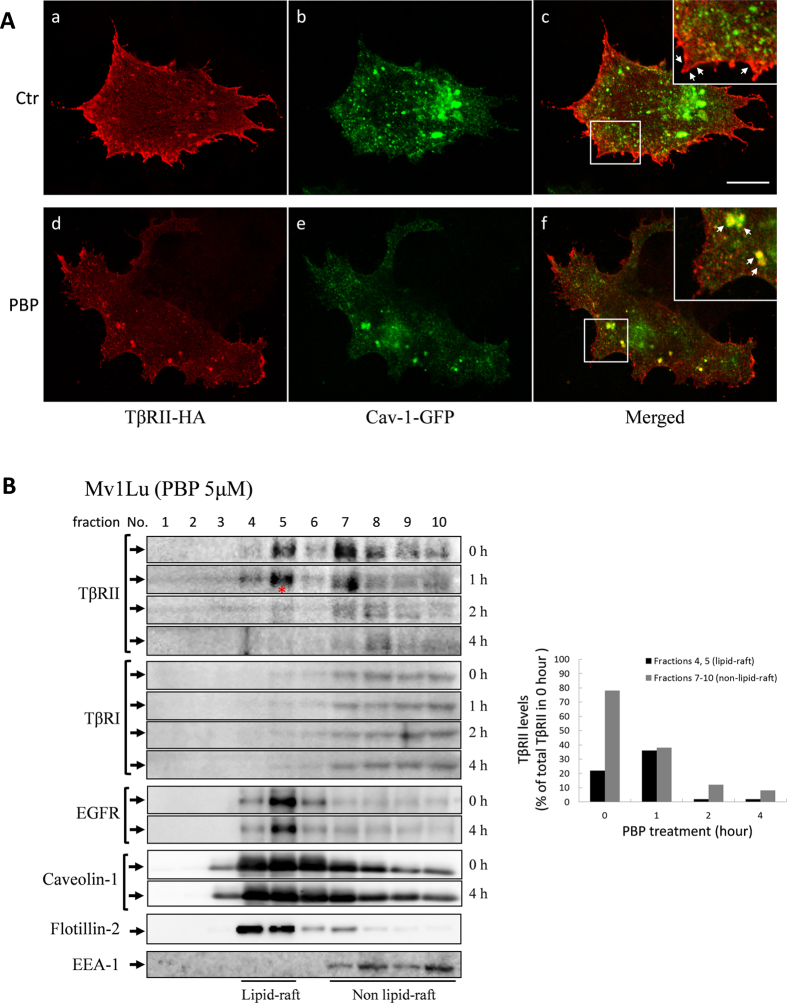
PBP induced the translocation of TβRII-HA from the plasma membrane to caveolin-1-positive cytoplasmic vesicles in Mv1Lu cells (**A**) and recruited TβRII from non-lipid raft microdomains to lipid rafts/caveolae for degradation (**B**). Mv1Lu cells transiently transfected with the plasmid expressing TβRII-HA (from Addgene) were treated with and without 5 μM PBP (panels Ad, Ae, and Af and panels Aa, Ab, and Ac, respectively) at 37 °C for 2 h. Cells were then analysed by performing indirect immunofluorescence staining with anti-HA (panels Aa and Ad) and anti-caveolin-1 antibodies (panels Ab and Ae). Merged staining is shown in panels Ac and Af. Before PBP treatment, TβRII-HA was primarily present on the plasma membrane and caveolin-1-GFP was primarily present in the cytoplasm of Mv1Lu cells. Arrowheads in the inset of panel Ac indicate TβRII-HA on the plasma membrane (red colour). PBP treatment reduced the levels of TβRII-HA on the cell surface and transferred TβRII-HA into caveolin-1-positive vesicles in the cytoplasm. Arrowheads in the inset of panel Af indicate the colocalisation (yellow colour) of TβRII-HA and caveolin-1 on the plasma membrane (panels Af); scale bar = 10 microns. (**B**) Mv1Lu cells were treated with 5 μM PBP at 37 °C for 0, 1, 2, and 4 h. Localisation of TβRII, TβRI, EGFR, caveolin-1, flotillin-2, and EEA-1 (early endosome antigen 1) in lipid rafts/caveolae and non-lipid raft microdomains in cells treated and not treated (control) with PBP were determined by performing sucrose gradient ultracentrifugation followed by immunoblotting with antibodies against TβRII, TβRI, EGFR, EEA-1, flotillin-2, and caveolin-1. Fractions 4 and 5, which mainly contained caveolin-1, represent the location of lipid rafts/caveolae (Lipid raft). Fractions 7, 8, 9, and 10 which contained EEA-1, represent the location of non-lipid raft microdomains (Non-lipid raft). Non-lipid raft contains small amounts of caveolin-1. This is due to the presence of mitochondria in these fractions^31^,^33^,^59^). The *symbol indicates the slightly increased amount of TβR-II in the fraction of cells treated with PBP for 2 h as compared with that in control cells. For longer treatments with PBP (2 h and 4 h), the closed arrow heads indicate the decreased amount of TβR-II in the fraction of PBP-treated cells as compared to that in control cells. The relative total amount of TβR-II in lipid rafts/cavelolae and non-lipid raft microdomains in control experiment (0 h) were taken as 100% (black bar + grey bar in 0 h). For example, the relative amounts of TβR-II in lipid-rafts in cells treated with PBP for 0. 1. 2. and 4 h were estimated to be 22%, 38%, 3%, and 2%, respectively; the relative amounts of TβR-II in non-lipid-rafts in cells treated with PBP for 0. 1. 2. and 4 h were estimated to be 78%, 40%, 13%, and 9%, respectively.

**Figure 9:**
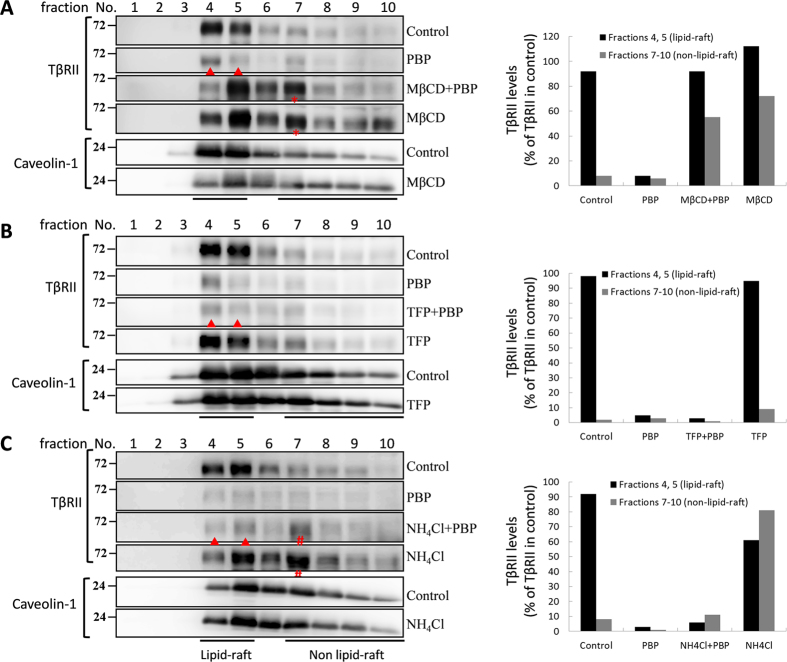
The effects of MβCD (**A**), TFP (**B**), and NH_4_Cl (**C**) in PBP-induced TβRII translocation and degradation in Mv1Lu cells. Mv1Lu cells were treated with 5 μM PBP with or without MβCD (A), TFP (**B**), and NH_4_Cl (**C**) at 37 °C for 4 h. Localisation of TβRII and caveolin-1 in lipid rafts/caveolae and non-lipid raft microdomains in cells treated and not treated (control) with PBP were determined by performing sucrose gradient ultracentrifugation followed by immunoblotting with antibodies against TβRII and caveolin-1. Representative of three experiments are shown. Fractions 4 and 5, which mainly contained caveolin-1, represent the location of lipid rafts/caveolae (lipid-raft). Fractions 7, 8, 9 and 10, which represent the location of non-lipid raft microdomains (Non lipid-raft). The closed arrow head indicates the decreased amount of TβR-II in the fraction of cells treated with PBP as compared with that in control cells. The * symbol indicates the increased amount of TβR-II in the fraction of MβCD-treated cells as compared to that in control cells. The #symbol indicates the increased amount of TβR-II in the fraction of NH_4_Cl-treated cells as compared to that in control cells. The relative amounts of TβR-II in the microdomains in treated cells were quantified by densitometry using caveolin-1 as an internal control. The relative total amount of TβR-II in lipid rafts/cavelolae (fractions 4 and 5, black bar) and non-lipid raft microdomains (fractions 7, 8, 9 and 10, gray bar) in control cells was taken as 100% (lipid-raft + non-lipid raft in control experiment). For example, the relative amounts of TβR-II in lipid rafts/caveolae (lipid raft) and non-lipid raft microdomains (non-lipid raft) in cells treated with PBP were estimated to be 4~8%, and 1~5%, respectively. The experiments in all three panels (Fig. 9A–C) were performed independently.

Previous studies have suggested that lipid rafts/caveolae induce proteasome-mediated degradation of TβRII in the absence of a ligand^26^. Therefore, we examined whether PBP-induced TβRII degradation was dependent on lipid rafts/caveolae. In this study, caveolin-1 and flotillin-2 were used as markers for lipid-raft/caveolae. Flotillins are topologically similar but unrelated in sequence to caveolins^44^. In fact, they were thought to be present in caveolae^45^ or to substitute for caveolae in cell types or tissues, such as leukocytes, which lack detectable caveolin-1^46^. Immunostaining assay results revealed that the overexpressed TβRII-HA was located on the cell surface and in cytoplasm. PBP treatment for 2 h markedly reduced the levels of TβRII-HA on the cell surface (Fig. 8Af versus Ac) and increased caveolin-1-GFP and TβRII-HA colocalisation (Fig. 8Af, as indicated by arrowheads). To corroborate these observations regarding PBP-induced TβRII translocation, we examined the effect of PBP on the subcellular localisation and degradation of TβRII in the Mv1Lu cells by performing sucrose gradient ultracentrifugation. In Fig. 8B, the results showed that TβRII was distributed in both lipid raft and non-lipid raft fractions in the control experiment (0 h); in the first hour after PBP treatment, TβRII in non-lipid raft fractions (fractions 7 to 10) not only slightly decreased, but also shifted to lipid-raft fractions (fraction 4 and 5) in the plasma membrane (marked with a red star), and it continued to turnover in prolonged treatment (2 h and 4 h). Conversely, PBP treatment induced neither translocation nor degradation of TβRI, EGFR, and caveolin-1 in this study. To further define the degradation route for PBP-induced TβRII turnover, we performed density gradient fractionation to determine the effects of inhibitors in PBP-induced TβRII translocation and degradation. In Fig. 9A, TβRII which found primarily in the lipid-raft fractions of Mv1Lu cells in control experiment and 4 hours PBP treatment induced TβRII degradation (Fig. 9A, denote as ▴). MβCD, a lipid-raft/caveolae disruptor, not only reversed PBP-induced TβRII degradation in lipid-raft but also moved the TβRII from lipid-raft to non-lipid raft fraction (Fig. 9A, denote as *, right graph for quantification). We also test whether clathrin-mediated endocytosis, another endocytic pathway for TGF-β receptor could confer PBP-induced TβRII turnover. In Fig. 9B, TFP (trifluoperazine), an inhibitor of clathrin-mediated endocytosis/recycling/lysosome route for TβRII, did not reverse TβRII turnover in any of the fractions (Fig. 9B, denote as ▴). In Fig. 9C, we use NH_4_Cl, a weak base that blocks lysosomal degradation by neutralizing proton accumulation in the process of lysosome maturation. NH_4_Cl does not prevent PBP-induced TβRII degradation in lipid-raft (Fig. 9C, denote as ▴). However, inhibition of lysosome maturation by NH_4_Cl treatment may cause accumulation of TβRII in pre-lysosomal compartments in high density fractions (Fig. 9C, denote as #) and slightly retard TβRII from PBP-induced degradation. It is noteworthy that only MβCD alter caveolin-1 partitioning between lipid-raft and non-lipid raft, which indicates that MβCD wreck caveolae and obstruct its function (Fig. 9A). Consistent with the preceding results (Figs 6 and 7C) of the present study, caveolae-mediated endocytosis inhibitor (MβCD) abolished PBP-induced TβRII degradation but not TFP and NH_4_Cl. These results suggest that PBP-induced TβRII degradation is through caveolae-mediated endocytosis.

## Discussion

In this study, the inhibitory effects of PBP on TGF-β signalling were characterised using Mv1Lu, A549, and NMuMG cells. This study also investigated the ability of PBP to induce the internalisation and turnover of TβRII; inhibit the migration of cells; and affect the expression of TGF-β-regulated proteins such as PAI-1, fibronectin, N-cadherin, vimentin, and E-cadherin. PBP is one of the most frequently used BFRs, extensively employed as an additive in resins and polyester polymers for improving their fire resistance. Other classes of BFRs, such as brominated bisphenols, may break down into PBP, which has higher bioavailability. Detailed information about the potential mechanisms underlying the biological and toxic effects of PBP is scarce. The results of the present study demonstrate, for the first time, that PBP inhibits TGF-β signalling by increases the clearance rate of TβRII from the cell surface, and by accelerating their turnover. The results also confirm our hypothesis that PBP promotes caveolae-mediated endocytosis of cell surface TβRII, resulting in the degradation of TβRII and subsequent termination of the signalling of TGF-β. These results are corroborated by the following findings. First, PBP inhibited all TGF-β responses examined in this study including Smad2 phosphorylation, PAI-1 promoter activation, EMT, and cell migration. Second, PBP treatment for 30 min reduced TβRII expression levels on the surface of the Mv1Lu cells by 58%, as determined by performing cell surface biotinylation (Fig. 5A). This reduction in cell surface TβRII expression is concurrent with drops in the total TβRII protein levels and TGF-β-induced cellular responses, suggesting that PBP suppresses TGF-β signalling by inducing the rapid internalisation and degradation of TβRII. The halogenated phenol 2,4,6-triiodophenol (TIP) is an analogue of PBP and has been used in this study. A recent series of experimental binding and computational studies have suggested that the TIP as an inhibitor of the ATPase activity of myosin VI^47^. Live cell image studies also suggested that TIP inhibits myosin VI-mediated vesicle secretion/recycling, with an IC_50_ of approximately 2 μM which is similar to PBP in this study^47^,^48^. However, TIP did not affect TGF-β signalling (Fig. 1A), which further implicate that PBP might sequester TβRII from cell surface by promoting TβRII internalization rather than inhibition of recycling. Therefore, additional studies will be necessary to characterize the binding sites and mechanism of PBP inhibition of the TβRII.

Ligand binding triggers TGF-β receptor endocytosis through clathrin- and caveolae-mediated pathways^19^,^49^,^50^,^51^,^52^,^53^. Clathrin-mediated endocytosis transfers receptors into an early endosome. Such internalised receptors are then either recycled to the cell surface or sent to the lysosomes for degradation. Caveolae-mediated endocytosis involving lipid rafts is a crucial trafficking pathway for TGF-β receptor internalisation and its ubiquitin-mediated degradation in the absence of a ligand^54^. Depletion of membrane cholesterol disrupts lipid rafts/caveolae, thus inhibiting caveolae-mediated endocytosis. Hence, we used clathrin-mediated endocytosis inhibitor TFP and cholesterol chelator MβCD to determine the endocytic pathway involved in PBP-induced TβRII degradation. Our results reveal that PBP-induced TβRII degradation was considerably blocked by MβCD, rather than TFP (Fig. 6). MβCD not only attenuated PBP-induced TβRII degradation but also reversed the inhibitory effect of PBP on TGF-β signalling including Smad2 phosphorylation and reporter gene activation (Fig. 7A and B). The results from cell surface labeling and sucrose gradient fractionation reveal that MβCD not only prevents PBP-enhanced caveolae-mediated endocytosis of cell surface TβRII (Figs 7C and 9A), but also moved the TβRII from lipid-raft to non-lipid raft fraction (Fig. 9A, denote as *). In contrast, TFP (trifluoperazine), an inhibitor of clathrin-mediated endocytosis/recycling /lysosome route for TβRII, neither changes TβRII localization nor reverses PBP-induced TβRII turnover (Figs 6A,D and 9B, denote as ▴). These results demonstrate that PBP induces TβRII degradation through caveolae-mediated endocytosis. Lysosomes are expected to degrade internalised proteins more efficiently at low pH levels because lysosomal hydrolysis typically requires acidic pH. Increasing lysosomal pH levels by adding weak bases such as NH_4_Cl and chloroquine can considerably reduce protein degradation in lysosomes^50^. However, our results reveal that PBP-induced TβRII degradation was attenuated after treatment with the proteasome inhibitor MG132 but not after treatment with the lysosomal inhibitor NH_4_Cl (Figs 6A,C and 9C, denote as ▴). In fact, NH_4_Cl, prevents lysosomal maturation by neutralizing proton accumulation and NH_4_Cl treatment causes accumulation of TβRII in pre-lysosomal compartments in high density fractions (Fig. 9C, denote as #) and slightly retards TβRII from PBP-induced degradation. Therefore, we conclude that PBP regulates the proteasomal degradation of TGF-β receptors through caveolae-mediated endocytosis. Previous studies described equal proteasomal degradation of both TβRI and TβRII through the ubiquitin-dependent (ubiquitin ligase Smurf2 mediated)^19^ or ubiquitin–independent pathway which is exclusively for TβRII^55^. Our data on PBP meets the later mechanism since PBP induces TβRII degradation without changing TβRI, EGFR, and caveolin-1 level; and the process is ubiquitin-independent. To exclude the possibility that PBP enhances TβRII degradation via ubiquitin-dependent pathway, we have tried to detect PBP-induced ubiquitination signal in endogenous TβRII or overexpressed TβRII-Flag, no ubiquitination signal was detected (Figure S5). Therefore, we suggest that PBP may possess a third mechanism of specific degradation exclusive for TβRII^55^. Wells *et al*. have shown different half-lives for TβRI and TβRII, which also echo with the concept that PBP induces distinct degradation mechanisms may exist to remove TβRII from cell surface^56^.

In addition to the inhibitory effects of PBP in TβRII turnover and TGF-β signaling, this study has elicited an important question about intracellular trafficking of TGF-β receptors and their degradation routes. It has long been recognized that TβRI and TβRII form hetero-complexes and co-translocated (or co-internalised) into intracellular compartments. However, we show that PBP selectively induces TβRII translocation and further degradation without affecting TβRI and other receptor such as EGFR (Figs 6A and 8B). In the future, additional studies should be performed to determine the targets of PBP in the endocytic machinery and TβRII degradation pathways. We will use SPR (Surface Plasma Resonance) to study interaction between PBP and TβRII or alternatively employ NMR to test PBP-TβRII interaction by observing the changes of ^1^H and ^13^C chemical shift. Although the direct target of PBP remains to be elucidated, it is possible that PBP directly binds TβRII to drive its internalization and degradation. It is also possible that PBP directs TβRII sorting by affecting its companion proteins follow by internalization. Interestingly, TβRII appears to be exclusively downregulated in several human cancers such as renal carcinomas and this reduction has been attributed to increased proteasomal degradation^55^,^57^,^58^. PBP might therefore be useful as a probe to understand how the altered dynamics of TβRII trafficking contributes to cancer.

Analyzing selective TGF-β-suppressing effects of PBDEs is outside the scope of the present study. However, there is an enormous body of evidence which demonstrates that the availability and function of TβRII is crucial determinants of TGF-β signaling and aberrant TGF-β responses are frequent in human diseases, such as cancer, fibrosis, inflammation, and cardiovascular disease. Therefore, the bioaccumulative and TGF-β-inhibitory properties of PBP observed in the present study suggest the potential effects of PBP and PBDEs in cancer development and TGF-β-relative diseases *in vivo*.

In conclusion, we found that PBP negatively regulated TGF-β signalling by enhancing TβRII degradation. The biochemistry approach revealed that PBP acts by stimulating clearance of TβRII from the cell surface through caveolae-mediated endocytosis and subsequent proteasomal degradation. However, additional *in vivo* studies are required to elucidate the potential targets and toxic effects of PBP. Considering these adverse effects of PBP, conducting a systemic assessment of the potential ecotoxic and biological effects of phenolic BFRs and relative compounds is imperative.

## Additional Information

**How to cite this article:** Chen, C.-L. *et al*. Pentabromophenol suppresses TGF-β signaling by accelerating degradation of type II TGF-β receptors via caveolae-mediated endocytosis. *Sci. Rep.* **7**, 43206; doi: 10.1038/srep43206 (2017).

**Publisher's note:** Springer Nature remains neutral with regard to jurisdictional claims in published maps and institutional affiliations.

## Supplementary Material

Supplemental Data
