# Supplementary material for: Pentabromophenol suppresses TGF-β signaling by accelerating degradation of type II TGF-β receptors via caveolae-mediated endocytosis
Source: Sci Rep. 2017 Feb 23;7:43206. doi: 10.1038/srep43206 (PMC5322341; doi:10.1038/srep43206)
Supplement: Supplemental Data [file srep43206-s1.pdf]

Supplementary Information

Pentabromophenol suppresses TGF-β signaling by accelerating degradation of type II TGF-β receptors through caveolae-mediated endocytosis

Chun-Lin Chen, Pei-Hua Yang, Yu-Chen Kao, Pei-Yu Chen, Chih-Ling Chung, and Shih-Wei Wang

Supplementary figures

Figure S1 : Cytotoxicity of PBP in A549, NMuMG, and Mv1Lu cells.

Figure S2: PBP inhibited TGF-β-induced Smad2 phosphorylation in NMuMG cells.

Figure S3: PBP did not affect RNA transcript levels of type I and type II TGF-β receptors.

Figure S4: MG132 did not reverse the inhibitory effect of PBP in TGF-β-induced Smad2 phosphorylation in Mv1Lu cells.

Figure S5: PBP degrades TβRII in an ubiquitin-independent manner.

Supplementary Material and Methods

MTT assay

A total of ~5000 cells were plated in 0.1 ml in 96-well flat bottom culture plates and then exposed to different concentration of PBP (final volume 0.2 ml per well, with or without 0.1% FBS). At the indicated times (6, 24, and 48 h), 20 μl of 5 mg/ml MTT solution in PBS were added to each well for 4 h. After removal of the medium, 170 μl of DMSO were added to each well to dissolve the formazan crystals. The absorbance at 540 nm was determined using a Biokinetics plate reader (Bio-Tek Instruments, Inc, Winooski, VT, USA). Triplicate wells were assayed for each condition were determined.

RNA isolation and RT-PCR

The procedurals for total RNA isolation and reverse transcription were described in our previous work (34). In brief, total RNA was isolated from treated cells using the TRIzol reagent according to manufacturer instructions. The target cDNA synthesis was performed using M-MLV Reverse Transcriptase and the synthesized cDNA was then amplified by Taq polymerase along with the following primers: TβR-II, (forward) 5'-TGCACATCGTCCTGTGGAC-3' and (reverse) 5'-GTCTCAAACTGCTCTGAAGTGTTC-3'; TβR-I, (forward) 5'-CGTGCTGACATCTATGCAAT-3' and (reverse) 5'-AGCTGCTCCATTGGCATAC-3'; GAPDH, (forward) 5'-GCATGGCCTTCCGTGTTC-3' and (reverse) 5'-GATGTCATCATACTTGGCAGGTTT-3'. Amplified DNA was analysed using agarose gel electrophoresis.

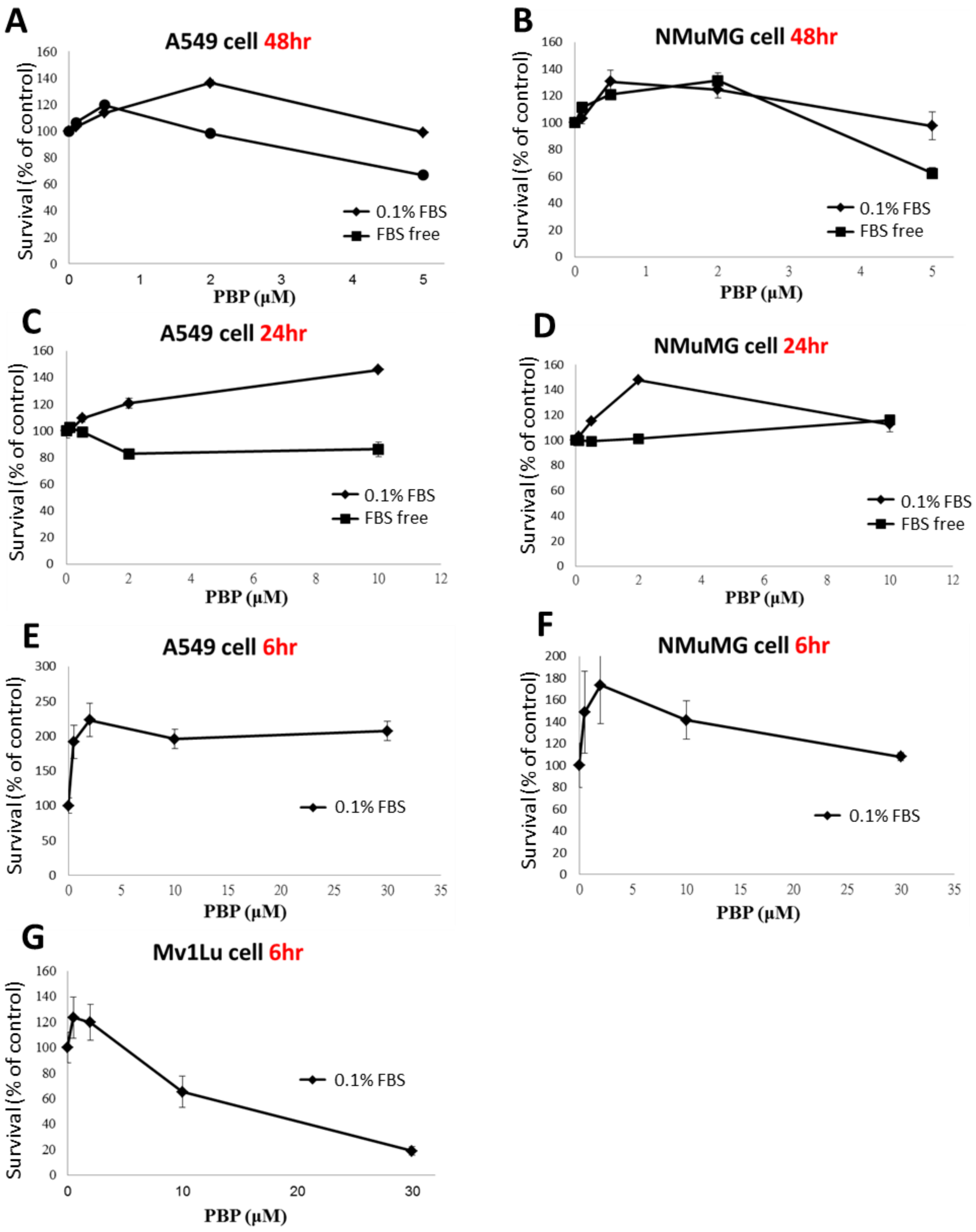

Supplemental data Figure S1. Cytotoxicity of PBP in A549, NMuMG, and Mv1Lu cells. Colorimetric MTT assays were employed to test short- and long-term toxicity of PBP. Cells were incubated with indicated concentration of PBP in the presence (closed diamond) or absents (closed square) of 0.1% FBS. All experiments were repeated three times, and data are expressed as mean ± SD.

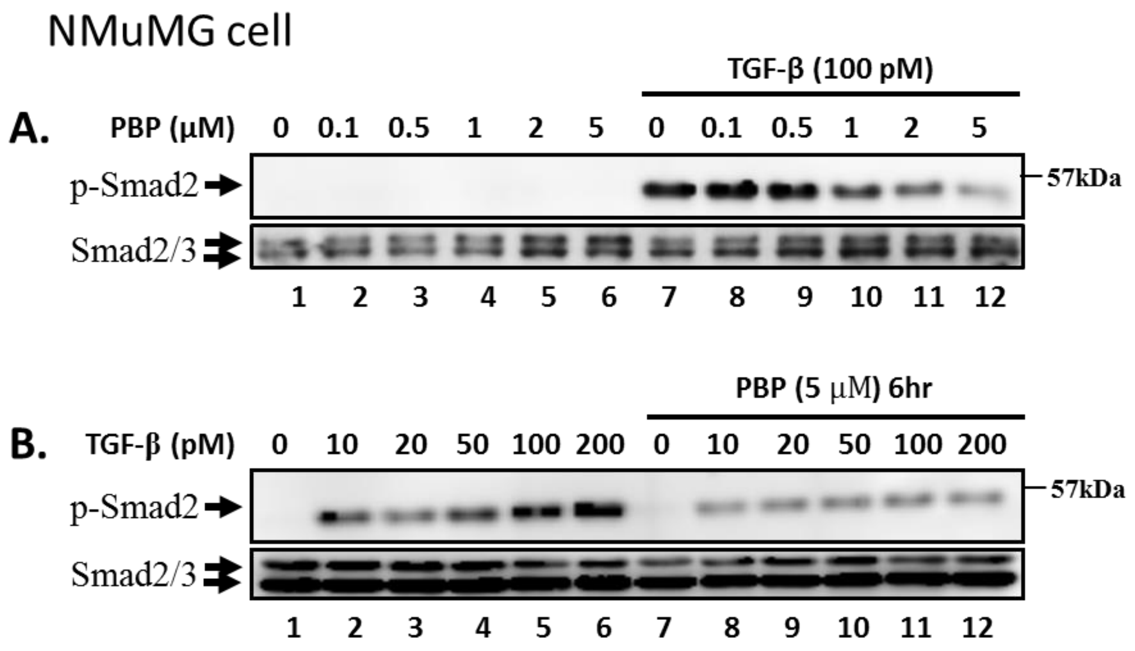

Supplemental data Figure S2. PBP inhibited TGF-β-induced Smad2 phosphorylation in NMuMG cells. NMuMG cells were cultured in low FBS (0.1%) DMEM and were pre-treated with increasing concentrations of PBP (0.1 to 5 μM) (A) or single concentration of PBP (5 μM) (B) for 6 h. After pre-treatment with PBP, the cells were stimulated with TGF-β (100 pM, or 10 to 200 pM) for 30 min. The cell lysates were resolved by performing western blotting to assess Smad2 phosphorylation. Smad2/3 served as an internal control.

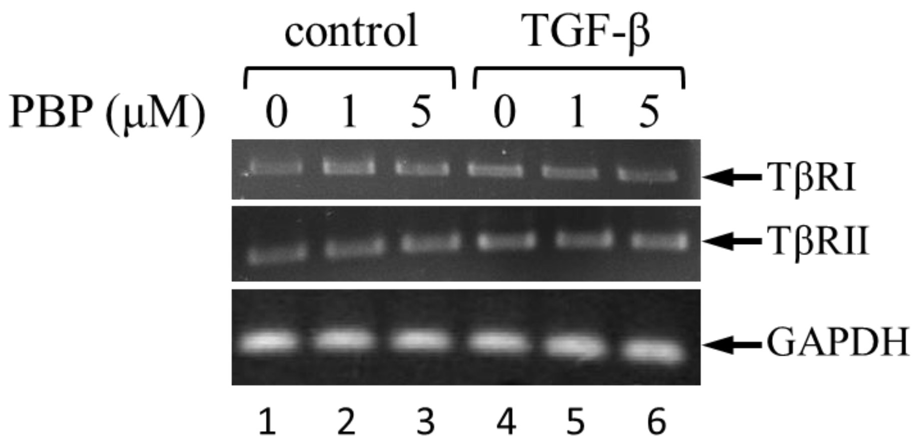

Supplemental data Figure S3. PBP did not affect RNA transcript levels of type I and type II TGF-β receptors in the Mv1Lu cell line. Mv1Lu cells were separately pre-treated with increasing concentration of PBP (0, 1, and 5 μM) for 1h and continued with TGF-β (100 pM) stimulation for 6 h. Low concentration of FBS (0.1%) was present all the experiments during PBP and TGF-β treatments. The procedurals for total RNA isolation and reverse transcription were described in Materials and Methods. In brief, total RNA was isolated from treated cells using the TRIzol reagent and the target cDNA synthesis was performed using M-MLV Reverse Transcriptase and the synthesized cDNA was then amplified by Taq polymerase along with the TβRI, TβRII, and GAPDH primers. It was revealed that with increasing PBP concentrations, the mRNA expression levels for TβRI and TβRII were not changed, suggesting the PBP-inhibited TβRII protein production is not due to the inhibition of TβRII mRNA production.

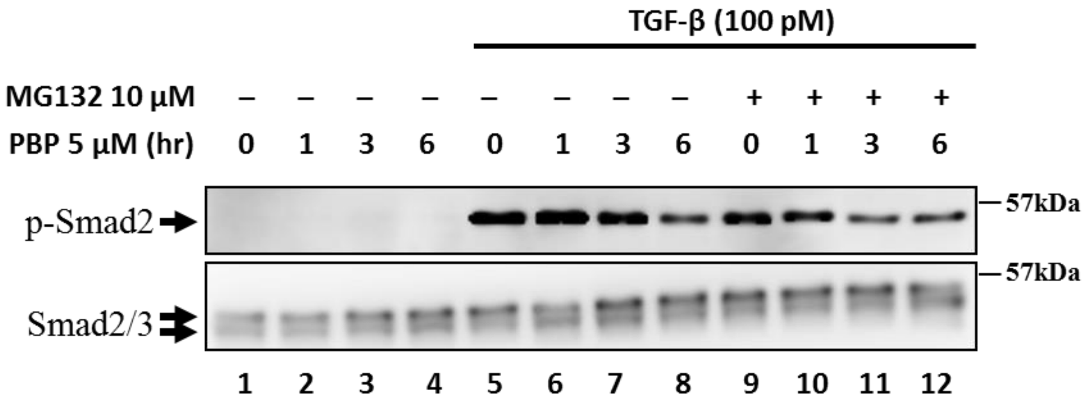

Supplemental data Figure S4. MG132 did not reverse the inhibitory effect of PBP in TGF-β-induced Smad2 phosphorylation in Mv1Lu cells. Mv1Lu cells were pre-treated with 5μM PBP for the time indicated with or without 10 μM MG132 follow by TGF-β stimulation (30 min). Cell lysates were resolved by performing western blotting to assess Smad2 phosphorylation. Smad2/3 served as an internal control. The result shows that MG132 treatment did not prevent PBP-inhibited TGF-β-induced Smad2 phosphorylation (Lanes 8 versus 12). Even MG132 alone slightly suppressed Smad2 phosphorylation (Lanes 5 versus 9). This implicated that MG132 may has “off target” effect that directly affect TGF-β signaling.

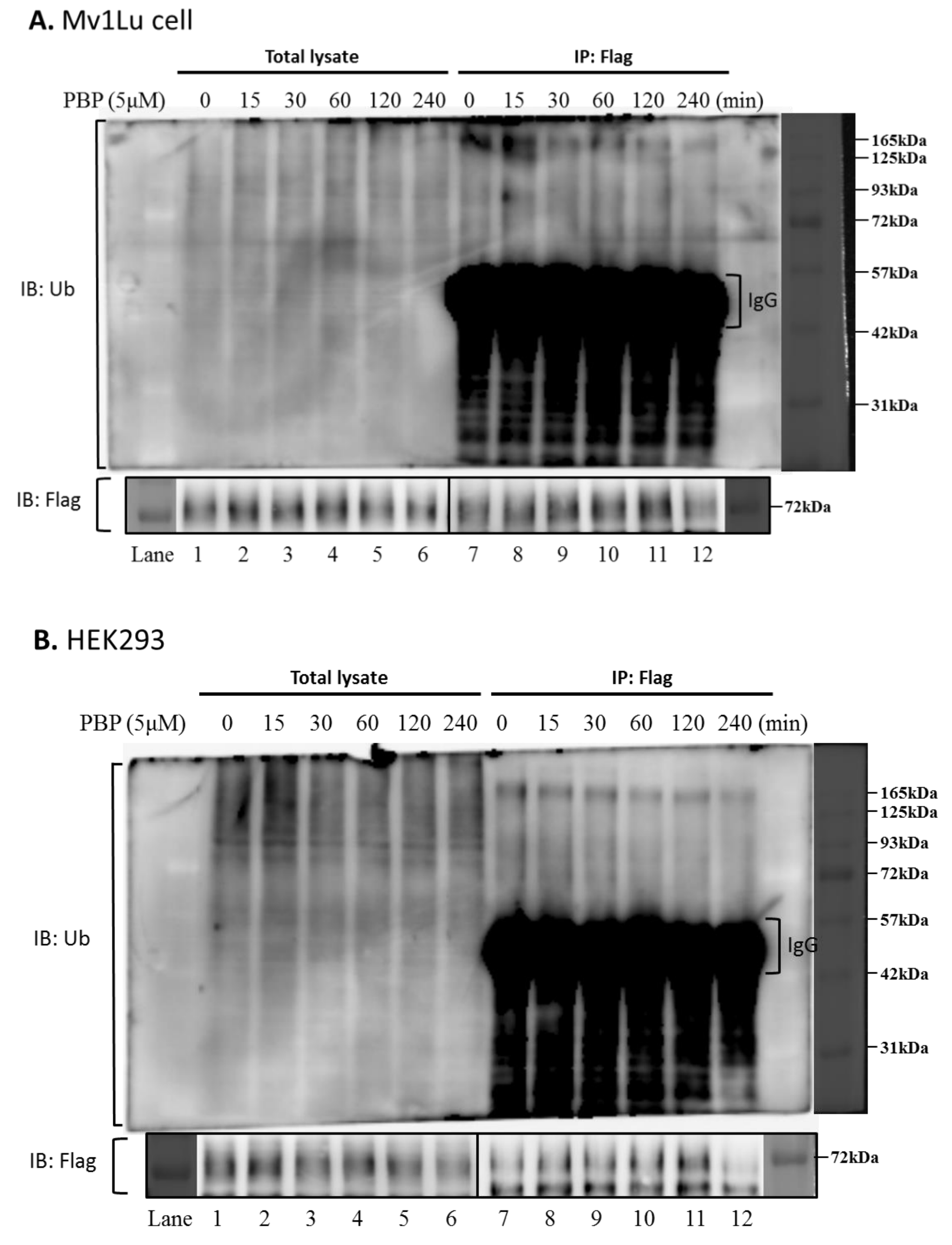

Supplemental data Figure S5. PBP degrades TβRII in an ubiquitin-independent manner. Mv1Lu (A) and HEK293T (B) cells were transfected with TβRII-Flag and treated with PBP for the indicated times. Proteasomal degradation was prevented by adding MG132 (10 μM) 4 hours before sample collection to enhance visualization of ubiquitinated proteins. A time course analysis of PBP treatment on overexpressed TβRII protein levels was performed to study ubiquitination of receptors after PBP treatment. There was no indication of PBP-mediated mono-ubiquitination of TβRII as no 10kD upward shift was observed for TβRII. Moreover, PBP did not increase overall ubiquitination (Lane 1 to 6) and did not lead to poly-ubiquitination of TβRII (Lane 7 to 12) (smear of size over 98kD). The membrane strips of protein markers (right side) were photographed separately in a 1:1 ratio to the blots. Black short lines indicate protein ladder sizes.
